# Supplementary material for: Impact of COVID-19-Related Lockdown on Delivery and Perinatal Outcomes: A Retrospective Cohort Study
Source: J Clin Med. 2022 Jan 30;11(3):756. doi: 10.3390/jcm11030756 (PMC8837050; doi:10.3390/jcm11030756)
Supplement: Supplementary file 1 [file jcm-11-00756-s001.zip › jcm-1522318-supplementary.pdf]

Table S1. Overall Caesarean delivery rates by period studied

	Overall CD			P1	P2
	**Pre-lockdown**	**Lockdown**	Post-lockdown		
Maternity A	184/**591** (31.1%)	178/**553 (32.2%)**	167/**594** (28.1%)	0.75	0.13
Maternity B	146/461 (31.7%)	137/**433 (31.7%)**	136/**447** (30.4%)	1	0.75
Maternity C	149/791 (18.8%)	157/782 (20.1%)	182/794 (22.9%)	0.57	0.18
Maternity D	185/735 (25.2%)	194/731(26.5%)	185/781 (23.7%)	0.58	0.23
Maternity E	167/*881 (18.9%)*	147/773 (19.0%)	186/803 (23.1%)	1	0.05
Maternity F	137/634 (21.6%)	133/557 (23.9%)	117/588 (19.9%)	0.38	0.12

Table S2: Operative Vaginal delivery rates by period studied

	Operative Vaginal deliveries			P1	P2
	**Pre-lockdown**	**Lockdown**	Post-lockdown		
Maternity A	74/**407** (18.2%)	85/**375 (22.7%)**	78/**427** (18.3%)	0.13	0.15
Maternity B	71/**315** (22.5%)	69/**296** (23.3%)	84/**311** (27.0%)	0.89	0.33
Maternity C	133/642 (20.7%)	131/622 (21.1%)	143/603 (23.7%)	0.97	0.34
Maternity D	61/550 (11.1%)	64/537 (11.9%)	82/596 (13.8%)	0,74	0.40
Maternity E	126/*714 (17.6%)*	95/626 (15.2%)	104/617 (16.8%)	0.25	0.48
Maternity F	54/496 (10.9%)	62/423 (14.7%)	80/471 (16.9%)	0.10	0.39

Table S3: Induction of labor rates by period studied

	Induction of labor			P1	P2
	**Pre-lockdown**	**Lockdown**	Post-lockdown		
Maternity A	163/590 (27.6%)	158/554 (28.5%)	181/594 (30.5%)	0.60	0.76
Maternity B	196/462 (42.4%)	172/433 (39.7%)	207/447 (46.3%)	**0.31**	**0.14**
Maternity C	177/673 (26.3%)	187/643(29.1%)	187/652 (28.7%)	0.18	0.58
Maternity D	191/735 (26.0%)	173/731(23.7%)	238/781(30.4%)	0.40	**0.01**
Maternity E	225/881 (25.5%)	185/773 (24.0%)	205/803(25.5%)	0.67	0.07
Maternity F	160/634 (25.2%)	175/557 (31.4%)	148/588 (25.2%)	0.6	0.03

Table S4: Severe postpartum hemorrhage rates by period studied

	Postpartum hemorrhage>1L			P1*	P2*
	**Pre-lockdown**	**Lockdown**	Post-lockdown		
Maternity A	8/589(1.4%)	10/553 (1.8%)	8/594 (1.3%)	0.71	0.53
Maternity B	8/461 (1.7%)	5/433 (1.1%)	7/447 (1.6%)	0.65	0.80
Maternity C	22/791 (2.7%)	22/782 (2.8%)	23/794 (2.9%)	1	1
Maternity D	41/735 (5.7%)	44/731 (6.0%)	52/781 (6.6%)	0.81	0. 61
Maternity E	21/881 (2.4%)	23/773 (3.0%)	26/803 (3.3%)	0.55	0.87
Maternity F	25/634 (3.9%)	15/557 (2.7%)	14/591 (2.4%)	0.35	0.88

Table S5: Maternal transfusion rates by period studied

	Transfusion			P1	P2
	**Pre-lockdown**	**Lockdown**	Post-lockdown		
Maternity A	4/590 (0.6%)	8/553 (1.4%)	3/593 (0.5%)	0.32	0.18
Maternity B	5/402 (1.2%)	5/375(1.3%)	3/387 (0.8%)	1	0.69
Maternity C	9/791 (1.1%)	9/782 (1.2%)	7/794 (0.9%)	1	0.78
Maternity D	8/735 (1.1%)	5/731 (0.7%)	7/781 (0.9%)	0.58	0.86
Maternity E	7/881 (0.8%)	11/773 (1.4%)	10/803 (1.2%)	0.32	0.93

Maternity F	4/634 (0,6%)	3/557 (0,5%)	6/588 (1,0%)	1	0,55

Table S6: Perineal tear rates by period studied

	**Perineal tear**				
	**Pre-lockdown**	**Lockdown**	Post-lockdown	P1	P2
Maternity A	10/411 (2.4%)	8/374 (2.4%)	11/430 (2.6%)	0 .97	0.87
Maternity B	1/467 (0.2%)	6/433 (1.4%)	3/446 (0.7%)	0.10	0.40
Maternity C	9/791 (1.1%)	9/782 (1.2%)	5/794 (0.7%)	1	0.56
Maternity D	10/734 (1.3%)	8/730 (1.1%)	18/782 (2.3%)	0.82	0.10
Maternity E	11/883 (1.2%)	10/775 (1.3%)		1	
Maternity F	5/504 (1.0%)	3/426 (0.7%)	9/473 (1.9%)	0.26	0.07
**stillbirth**					
	Before lockdown	During lockdown	After lockdown	P1	P2
Maternity A	3/593 (0.5%)	0/553 (0%)	3/591 (0.5%)	0.27	0.27
Maternity B	2/461 (0.4%)	5/433 (1.1%)	3/384 (0.8)	0.65	0.96
Maternity C	10/791 (1.2%)	6/782 (0.8%)	2/794 (0.2%)	0.64	0.27
Maternity D	6/734 (0.8%)	1/730 (0.1%)	4/782 (0.5%)	0.13	0.41
Maternity E	2/883 (0.2%)	5/775 (0.6%)	1/804 (1.2%)	0.35	0.20
Maternity F	0/634 (0%)	3/557 (0 .5%)	2/588 (0.3%)	0.20	0.95
